# Current Sample Preparation Methodologies for Determination of Catecholamines and Their Metabolites

**DOI:** 10.3390/molecules27092702

**Published:** 2022-04-22

**Authors:** Nian Shi, Xinmiao Bu, Manyu Zhang, Bin Wang, Xinli Xu, Xuezhong Shi, Dilshad Hussain, Xia Xu, Di Chen

**Affiliations:** 1 Physics Diagnostic Division, The First Affiliated Hospital of Zhengzhou University, Zhengzhou 450052, China; 13503823257@163.com; 2 Key Laboratory of Targeting Therapy and Diagnosis for Critical Diseases of Henan Province, School of Pharmaceutical Sciences, Zhengzhou University, Zhengzhou 450001, China; bxm155144@163.com (X.B.); zy2232631057@163.com (M.Z.); 18592059931@163.com (B.W.); xxl17837413765@163.com (X.X.); 3 College of Public Health, Zhengzhou University, Zhengzhou 450001, China; xzshi@zzu.edu.cn; 4 HEJ Research Institute of Chemistry, International Center for Chemical and Biological Sciences, University of Karachi, Karachi 75270, Pakistan

**Keywords:** catecholamines, sample preparation, chemical derivatization, chromatography

## Abstract

Catecholamines (CAs) and their metabolites play significant roles in many physiological processes. Changes in CAs concentration in vivo can serve as potential indicators for the diagnosis of several diseases such as pheochromocytoma and paraganglioma. Thus, the accurate quantification of CAs and their metabolites in biological samples is quite important and has attracted great research interest. However, due to their extremely low concentrations and numerous co-existing biological interferences, direct analysis of these endogenous compounds often suffers from severe difficulties. Employing suitable sample preparation techniques before instrument detection to enrich the target analytes and remove the interferences is a practicable and straightforward approach. To date, many sample preparation techniques such as solid-phase extraction (SPE), and liquid-liquid extraction (LLE) have been utilized to extract CAs and their metabolites from various biological samples. More recently, several modern techniques such as solid-phase microextraction (SPME), liquid–liquid microextraction (LLME), dispersive solid-phase extraction (DSPE), and chemical derivatizations have also been used with certain advanced features of automation and miniaturization. There are no review articles with the emphasis on sample preparations for the determination of catecholamine neurotransmitters in biological samples. Thus, this review aims to summarize recent progress and advances from 2015 to 2021, with emphasis on the sample preparation techniques combined with separation-based detection methods such capillary electrophoresis (CE) or liquid chromatography (LC) with various detectors. The current review manuscript would be helpful for the researchers with their research interests in diagnostic analysis and biological systems to choose suitable sample pretreatment and detection methods.

## 1. Introduction

Catecholamines (CAs), a class of neurotransmitters including dopamine (DA), norepinephrine (NE), and epinephrine (E), are circulating hormones synthesized by the phenylalanine-tyrosine metabolic pathway [1,2]. The levels of CAs along with their metabolites have significant correlations with the occurrence and development of many types of diseases, such as obesity [3], depression [4], Alzheimer’s disease [5], Parkinson’s disease [6,7], and phaeochromocytoma [8]. Different metabolites are associated with these catecholamines. 3,4-Dihydroxyphenylacetaldehyde (DOPAL) and catecholaldehyde, which is detoxified by aldehyde dehydrogenase (ALDH), are associated with dopamine. Similarly, metanephrines include normetanephrine and metanephrine, homovanillic acid (3-methoxy-4-hydroxyphenylacetic acid) and vanillylmandelic acid (3-methoxy-4-hydroxymandelic acid) are some other examples of metabolites produced from epinephrine, norepinephrine, and dopamine [9]. Therefore, the accurate quantification of CAs and their metabolites in biological samples is of great significance, especially in early clinical diagnosis, in-depth understanding of pathology, and contribution to developing better treatments.

**Figure 1:**
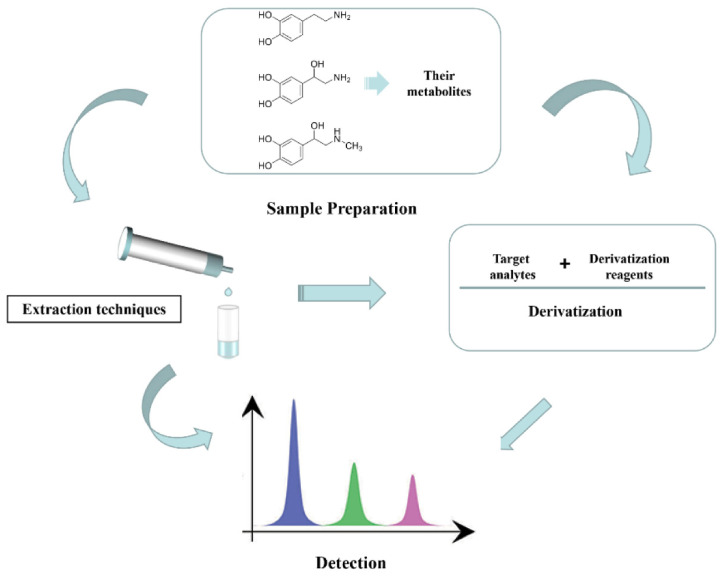
The analysis process for the determination of CAs and their metabolites.

Up to date, various detection techniques including sensing methods [10,11,12], spectroscopic methods [13,14], and chromatographic methods [15,16,17,18] have been utilized for the detection of endogenous CAs and their metabolites. Among these methods, separation-based detection techniques such as liquid chromatography (LC) and capillary electrophoresis (CE) have become the primary and most widely used techniques due to their unique characteristics of separating CAs from numerous co-existing interferences, exhibiting excellent specificity, precision, and reproducibility. LC or CE coupled with various detectors including ultraviolet (UV) [8], fluorescence (FLD) [19], electrochemical detection (ECD) [20,21], and mass spectrometry (MS) [22,23] all have been used for detecting CAs and their metabolites (Figure 1).

Although separation-based detection techniques such as LC or CE can separate target analytes from biological interferences based on the property differences to some extent, some practical problems still exist in the direct analysis of complex biological samples. Due to the extremely low concentrations of endogenous CAs, the sensitivity of direct detection is usually insufficient to meet the requirement of measurement [24]. The complex biological matrix may also result in the co-elution of interferences together with the target analytes, thus decreasing the detection specificity and precision. Besides, the sample morphology may not be compatible with the used detection techniques. Therefore, suitable sample preparations before instrument detection are always needed.

For the present, various sample preparation techniques have been developed. Although many determination methods for catecholamine neurotransmitters have been concluded in some reviews in recent years [11,13,14,25,26], a comprehensive summary focused with the emphasis on sample preparations for the determination of catecholamine neurotransmitters in biological samples is missing. Thus, here we present a comprehensive overview of the progress and advances of sample preparation methods for pretreatment of samples for CAs detection from 2015 to 2021, and detailed analytical information on these successful methods is also provided.

## 2. Sample Preparation

Normally, conventional liquid–liquid extraction (LLE), solid-phase extraction (SPE) technique, and chemical derivatization are regularly employed in bio-sample preparation. To decrease the sample volume required, the cost and time consumption, the trend in current sample preparation is moving toward environmental friendliness, miniaturization, simplicity, and automation. Hence, many novel sample preparation techniques have been developed, including solid-phase microextraction (SPME), liquid–liquid microextraction (LLME), dispersive solid-phase extraction (DSPE), and chemical derivatizations based on newly synthetic derivatization reagents. A brief description of these sample preparation techniques is given in the following sections, with relevant applications in which they are used for the detection of CAs and their metabolites in different matrices.

### 2.1. Liquid–Liquid Extraction-Based Approaches

Liquid–liquid extraction (LLE) is one of the oldest sample preparation methods, and it is based on solubility differences to implement a transfer of target analytes from the aqueous sample into another immiscible liquid phase [27]. The pH may also affect the distribution of the solute in both phases because the CAs containing phenol hydroxyl and amino are amphoteric substances. So solvent polarity and pH of the aqueous phases are the major factors to be considered for the analysis of CAs and their metabolites. Although the use of LLE alone provides a good purification effect as a non-specific method, the application of LLE has become limited due to the complexity of the CAs and their metabolites. Other sample preparation procedures are often combined with LLE in the detection of CAs and their metabolites. A few newly developed methods applying the LLE technique in combination with derivatization have been published on the determination of CAs and their metabolites [28]. Zhao et al. [29] designed and synthesized derivatization reagent 4′-carbonyl chloride rosaline (CCR) for simultaneous labeling of amino and hydroxyl groups in CAs and their metabolites. LLE was employed to extract the CCR-derivatives from the aqueous samples into the 4-bromoanisole, which could easily remove complex biological matrices and excess derivatization reagent.

Nevertheless, conventional LLE exhibits some shortcomings, such as the use of large sample volumes and toxic organic solvents (ethyl acetate, dichloromethane, chloroform, and carbon tetrachloride); these drawbacks make LLE time-consuming, laborious, and environmentally unfriendly. To overcome the disadvantage of LLE, liquid-phase microextraction (LPME), a new extract technique based on LLE is developed in 1996 [30]. LPME possesses fairly obvious advantages compared with liquid–liquid extraction like miniaturization, simplicity, rapidity, and environmental friendliness. So far, LPME has evolved into various forms such as dispersive liquid–liquid microextraction (DLLME) [29,31], single-drop microextraction (SDME) [32], ultrasound-assisted liquid–liquid microextraction (UA-LLME), hollow fiber liquid-phase microextraction (HF-LPME) [33], and headspace liquid-phase microextraction (HS-LPME) [34]. Recently, these various types of LPME methods have been successfully used for the separation and preconcentration of CAs and their metabolites in biological samples. Konieczna et al. [35] combined dispersive liquid–liquid microextraction (DLLME) with liquid chromatography coupled to mass spectrometry (LC-MS) to develop a simple and sensitive method for the determination of neurotransmitters in human urine samples. A mixture of ethanol (dispersion solvent) and dichloromethane (extraction solvent) was quickly injected into a human urine sample to form a turbid solution in an Eppendorf tube. After centrifugation, a simple clean-up process was realized and this method also exhibits good linearity and sensitivity. For aqueous biological samples, nonpolar or less polar water-immiscible organic solvents organic solvent is usually used as extraction solvent. It is difficult to extract CAs and their metabolites from the aqueous solution due to the low distribution coefficient of hydrophilic analytes in the organic solvent. Therefore, some novel strategies have been proposed to solve this predicament. Jiang et al. [36] attempted to add various carriers into the organic phase for improving the extraction efficiency of analytes, and analytes were effectively purified using negligible volumes of extracting solvent followed by centrifuging and collecting the precipitated phase by solidifying the aqueous stripping phase.

### 2.2. Solid-Phase Extraction (SPE)

Solid-phase extraction (SPE) is the most commonly employed method for the separation and concentration of target analytes. The principle of SPE is through retaining compounds of interest on solid-phase sorbents, and the purpose of purification of analytes is subsequently carried out by selective elution. In comparison to LLE, there are several distinct advantages, such as higher quantitative recovery, use of less organic solvents, greater flexibility due to the use of different sorbents, and greater possibility of automation [37]. Applications of SPE techniques for preconcentration of CAs and their metabolites in biological samples are summarized in Table 1.

Among a variety of sorbents, weak cation exchange (WCX)-based sorbents are preferred due to their improved extraction efficiency and excellent repeatability for the extraction of CAs and their metabolites [38,40]. In addition, reversed-phase hydrophobic- hydrophilic balanced (HLB) based sorbents have been also widely used including Strata-X-CW, Bond-Elut Plexa [39,43,44,46]. These sorbents based on WCX and HLB are all in mixed-mode SPE for the isolation of CAs and their metabolites, which has good retention for polar and non-polar compounds in water and can stay stable in wide pH ranges. Therefore, these developed methods using sorbents based on WCX and HLB, and easily obtained satisfactory linearity and recovery. Along with the increasing requirement for CAs detection, the trend in recent years is miniaturization, simplification, and automation. In SPE, many unusual changes also take place. Various novel product forms are created, including well-plates [41], and discs that differed from conventional column cartridges. Li et al. used a 96-well HLB microplate to accomplish a simple extraction of monoamine neurotransmitters from complex urine [47]. The extraction process has the significant characteristic of miniaturization and high throughput. The sorbent is the key factor affecting the SPE performance, some highly selective sorbents are designed and synthesized for preconcentration of CAs and their metabolites. Hou et al. [8] utilized the affinity between cis-diol-containing CAs and boric acid to prepare boronate-modified hollow dummy template imprinted polymers (B-hDIPs) for isolating CAs from urine samples, and extremely high extraction efficiency is obtained due to covalent bonding. The extraction of CAs in human urine was optimized using electrospun composite fibers functionalized with 4-carboxybenzo-18-crown-6 ether modified XAD resin and polystyrene by Chen et al. [45], and they packed this composite fiber into 96-well columns and achieved a miniaturization analysis of CAs. Moreover, several attempts have also been carried out about automated analysis of CAs and their metabolites [42]. This SPE mode has the advantage of using less sample and solvents volume, avoiding intensive labor, and is also the development trend of sample analysis in the future.

**Figure 2:**
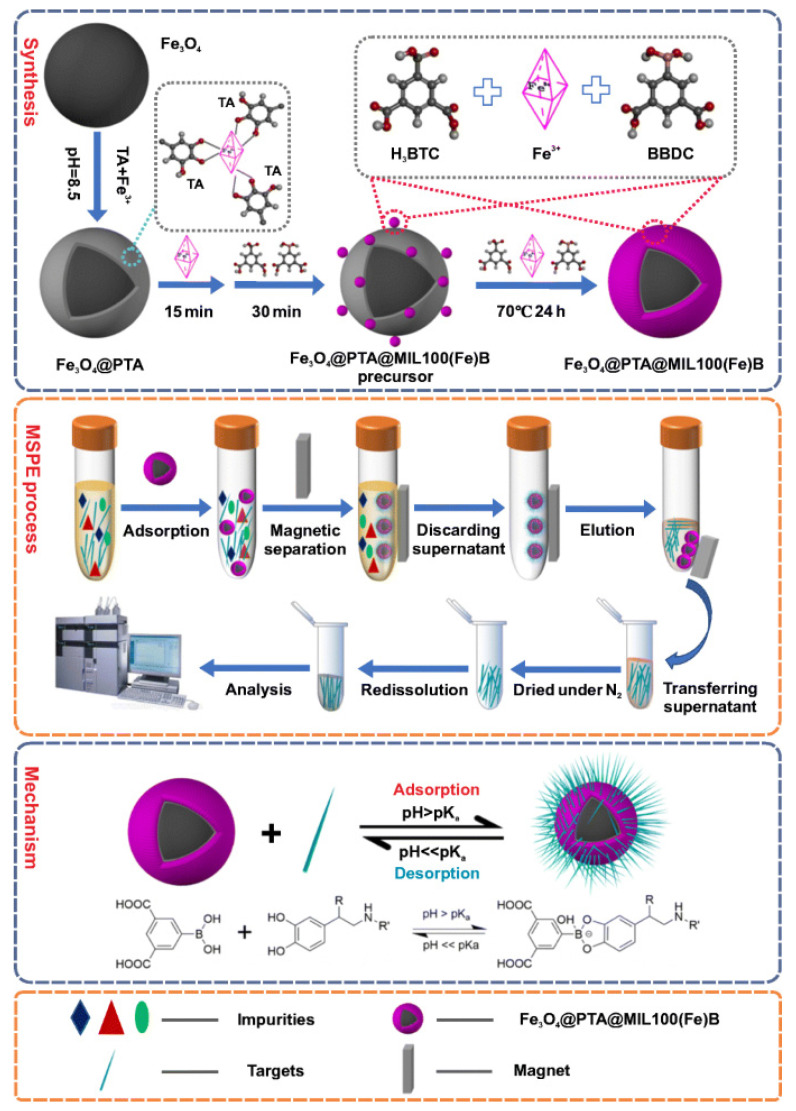
Synthesis mechanism and extraction of CAs by Fe_3_O_4_@PTA@MIL-100(Fe)-B [48].

Metal-organic frameworks (MOFs) are a fascinating class of organic–inorganic hybrids with unique properties including high surface area, regular three-dimensional structure, high porosity, and tunable surface chemistry. MOFs are being used as solid-phase extraction sorbents for the preconcentration and enrichment of biomolecules from the different complex samples [51,52]. Xing et al. [48] used polytannic acid (PTA) modified borate-functionalized metal-organic framework for the extraction and preconcentration of CAs and their metabolites from human urine samples. MOFs were prepared by metal-ligand-fragment co-assembly strategy and further modified with magnetic iron oxide nanoparticles to form Fe_3_O_4_@PTA@MIL-100(Fe)-B and applied as magnetic solid-phase extraction (MSPE) sorbents (Figure 2). For screening, the variable for extraction efficiency of the modified MOFs was investigated by Plackett–Burman design. Optimal extraction conditions were selected by Box–Behnken design. HPLC coupled with fluorescence detection was used for the analysis of catecholamines. Although the proposed method showed promising efficiency, the construction of such a highly ordered network is challenging. The stability of MOFs in harsh experimental conditions also hinders their practical applications.

In a similar approach, amino-functionalized polyhedral oligomeric silsesquioxanes (POSS-8NH2) were constructed by Wei et al. and further attached to polydopamine-coated magnetized graphene oxide via covalent modification [49]. The prepared sorbent (magGO@POSS-BA) contained cubic boronic acid and showed significantly higher selectivity for CAs due to the presence of cis-diol selective boronate affinity. The cage-like structure of POSS enhanced the surface area for better adsorption and acted as a building block for the composite formation. Although the material showed an extraordinarily high capacity for CAs in urine samples, the cost of synthesis is very high. Moreover, the adsorbent showed better selectivity for ortho-phenols.

A packed fiber solid-phase extraction approach is also reported recently for the detection of CAs. Electrospun poly crown ether composite was applied to selectively isolate CAs from urine samples with high reproducibility and precision [50]. The proposed online PFSPE-HPLC approach minimized the sample loss, decreased the operational errors, and improved the sensitivity of the analysis. The proposed approach is simple and rapid, one limitation is the selectivity in complex biological samples.

### 2.3. Dispersive Solid Phase Extraction/Microextraction (DSPE/DSPME)

Dispersive solid-phase extraction (DSPE) is a rapidly developing extraction technique since it was introduced in 2003 by Anastassiades and coworkers [53]. It isolates analytes from the liquid matrix by using a solid sorbent, which is directly dispersed into the sample solution. The adsorption process can be completed quickly by assisting by means of vortex, ultrasounds, etc. Then, the sorbent particles adsorbed analytes are separated by centrifugation, filtration, or magnetic attraction [54]. Finally, it needs to choose a suitable elution solution and elute the analytes. The elution solution can be directly injected into the detection instrument, or evaporated and reconstituted into a minor volume to achieve preconcentration and purification of analytes. DSPE as a highly developed form of SPE has attracted a lot of interest from analysts for the clean-up of CAs and their metabolites because of its unique superiority compared to SPE, such as being rapid, cheap, effective, and convenient [55]. Applications of DPSE techniques for preconcentration of CAs and their metabolites in different biological samples are summarized in Table 2.

Magnetic nanoparticles with surface modification are a promising adsorbent material for the separation of CAs and their metabolites [19,57]. Its nanoscale size can provide a relatively large specific surface area to obtain more active sites. Furthermore, there have also been studies using hollow-structured nanoparticles to make adsorbents larger [60]. Its magnetism makes it easy to separate it from the sample solution with the assistance of magnets. Meanwhile, the researchers also attempt to make the adsorbents possess more excellent selectivity by modifying various groups on the surface of magnetic nanoparticles [56,58]. For these above reasons, the DSPE procedure of the CAs detection has been greatly upgraded. In addition to this, the application of carbon nanotube for the detection of CAs and their metabolites has been reported by Ma et al. and Murtada et al. [55,59].

**Figure 3:**
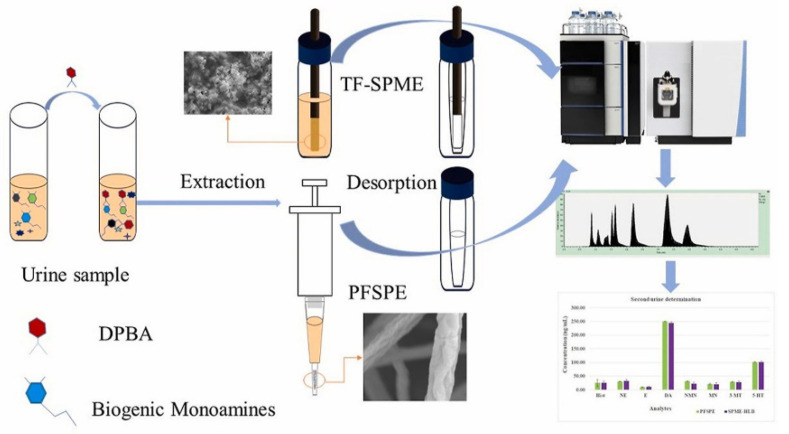
Determination of free biogenic monoamines and their metabolites in urine using thin-film solid-phase microextraction [61].

Biogenic monoamines are a class of catecholamines commonly used for the diagnosis of catecholamine-producing tumors. So, it is clinically significant to design novel methods for the detection of biogenic monoamines from biological samples. For this purpose, Pawliszyn et al. [61] proposed a strategy based on two different pretreatment methods: packed fibers solid phase extraction (PFSPE) and thin-film solid-phase microextraction (TF-SPME). The main goal of the study was to accurately quantify the metabolites of biogenic monoamines (epinephrine, norepinephrine, 3-methoxytyramine, dopamine, serotonin, normetanephrine, histamine, and metanephrine) in urine samples. Extraction was carried out via the thin-film blade format SPME method using a hydrophilic–lipophilic balance (HLB) coating. For PFSPE polycrown ether (PCE) composite nanofiber was used for comparison, and an extraction recovery of 35.7–74.8% was obtained under optimal conditions. TF-SPME method showed better performance than the PFSPE method, for the simultaneous determination of eight metabolites (Figure 3).

Molecularly imprinted polymers (MIPs) are used as a dispersive solid-phase extraction sorbent before UHPLC/MS/MS for simultaneous quantitative analysis of CAs and their metabolites. MIPs are highly selective for specific molecules, so they are commonly used in biological analysis. Podjava et al. [62] proposed a combined non-covalent and semi-covalent imprinting approach using different types of polymers and crosslinkers. The imprinted polymers showed good imprinting factors ranging from 3.1 to 5.6, indicating high selectivity of the analysis. In addition, the proposed method efficiently distinguished between methylated CAs and non-methylated CAs. Although MIPs-based extraction strategies are highly efficient and selective for particular types of analytes, the availability of monomers and cross-linkers for imprinting is a huge challenge. In addition, the cost of imprinting polymers is usually very high, limiting their practical applications.

In a recent approach, borated zirconia is used for the extraction of plasma catecholamines, followed by LC-MS/MS detection. Borated zirconia has an inherent affinity toward cis-diol compounds and it forms a five or six-membered reversible cyclic ester in basic conditions, capturing the cis-diol compounds and then releasing them in acidic conditions. The working principle of this method is simple and well-known and the sorbent efficiently captured dopamine (DA), epinephrine (E), and norepinephrine (NE) from the plasma samples of healthy volunteers [24].

### 2.4. Solid-Phase Microextraction (SPME)

SPME was first proposed in 1989 as an alternative to conventional extraction techniques and has been progressively favored by analysts in recent years [63]. It involves equilibrium between analytes and the fiber coating and followed by desorption using the suitable elution solution. The SPME procedure has a distinct features compared to other extraction techniques, including the simplicity of operation, use of small sample volumes, solvent-free extraction, and easily achieved automation. The main arrangements of SPME can be performed into fiber SPME and in-tube SPME. With the development of microextraction technology, other new forms of SPME have also been proposed: thin-film, in-tip, and in-needle microextraction, rotating disk sorptive extraction (RDSE), and capillary microextraction. SPME can overcome the defects of traditional solid-phase extraction techniques and exhibit high performance, and it has been successfully introduced in many fields of food, medicine and hygiene, biochemistry, toxicology, and forensic medicine.

**Figure 4:**
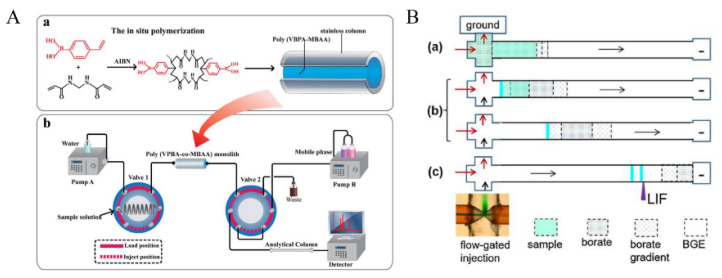
Schematic diagram of the online micro-solid-phase extraction system, reprinted from [70,71]. (**A**) The in-situ polymerization of monolithic column (**a**), schematic diagram of the online micro-solid-phase extraction system coupled to HPLC (**b**). (**B**) Schematic procedures showing on-line preconcentration and separation. (**a**) Sample injection; (**b**) focusing; and (**c**) separation.

For analysis of CAs and their metabolites, methods using coated fiber have been described, and many researchers put a significant effort into the development and optimization of these fibrous materials over the years. Recently, the fiber SPME has been innovated by Monteleone et al. [64]. The performance of five SPME fibers was evaluated, and the optimal one is finally determined to be the polyacrylate fiber. The fiber coated with the suitable organic polymer can extract and enrich derivatives of CAs and their metabolites from complex urine matrices by immersing them into the sample vial. Using such an easy procedure, an accurate assay of analytes can be completed. However, fiber SPME exhibits some restrictions, especially in bio-analytical laboratories, involving fragile properties or its life reduced at high salt concentrations. Hence, in-tube SPME gets more preference by degrees and become a universal extraction technique, since the open tubular fused silica capillary used for in-tube SPME can tune the column length and the thickness of extractant coating, and the analytes can be eluted by appropriate desorption solution and directly injected into CE or HPLC system after repeated extraction through an internally coated capillary. The in-tube SPME methods employing long-chain ionic liquids based on imidazole [65] and phenylboronic acid [66,67] for adsorbing CAs and their metabolites have been described. Initial in-tube SPME involves mainly offline applications [68,69], but some attempts for detection of CAs and their metabolites have been also taken to accomplish the convenient automation of the extraction process with online applications increasingly possible. Automated in-tube SPME not only reduces human errors caused by manual manipulation (Figure 4) but also provides significant gains in sensitivity and accuracy compared to manual off-line techniques [70,71]. Meanwhile, solvent-free operation and high throughput are also distinctive traits of automated in-tube SPME. However, these methods involved the application of automated in-tube SPME to date and have been combined with laboratory-made devices only, which makes the popularization of automated in-tube SPME limited in routine bio-analysis.

Microextraction by packed sorbent (MEPS) is a miniaturized form of solid-phase microextraction and it is commonly used these days for the extraction of a variety of compounds including drugs and metabolites. MEPS involves directly integrated packing into the syringe, and sample introduction does not require a separate robot. Another advantage of MEPS is that more than 100 plasma or urine samples could be analyzed by a single MEPS bed [72]. Xin et al. [73] designed a simple, rapid, and cost-effective method for the quantitative determination of CAs and their metabolites from urine samples. The designed method was semi-automated microextraction involving a digitally controlled syringe, before LC-MS/MS analysis. Different types of extraction sorbents including a polar enhanced polymer (PEP), cation-exchange (CX) and C18, and porous graphitized carbon (PGC) were tested. The whole analysis was completed within 4 min. The precision of the analysis was ≤12.8%, recoveries were 89–109.5%, and linear ranges were 0.167–686 ng/mL. The designed method showed comparable efficiency for PPGLs with commercial biochemical tests.

**Figure 5:**
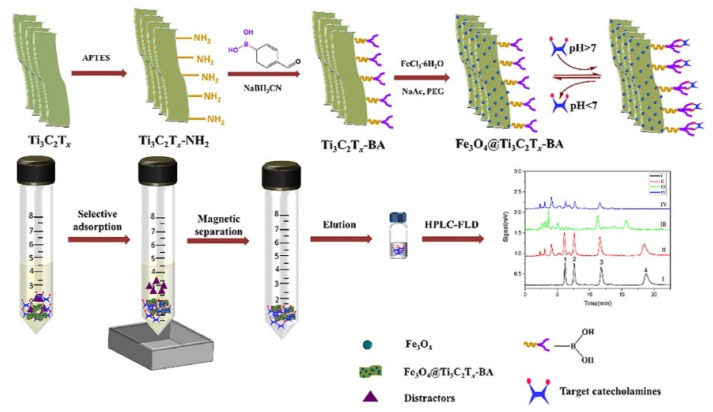
Magnetic borate-modified MXene composite for the extraction of CAs [74].

MXenes are a recent fascination in material science research, exhibiting some exciting properties. Magnetic boronic acid-modified Ti_3_C_2_T*x* composites are reported for the extraction of CAs with high selectivity and specificity, as shown in Figure 5 [74]. Due to the unique 2D layered structures of the sorbent, 319.6 μmol/g of dopamine is absorbed within 2 min, which facilitated the molecular transport and shortened the diffusion path. Fast adsorption is also attributed to the van der Waals forces, π–π stacking, hydrogen bonding, multilayer adsorption, and the synergetic interactions of borate affinity. The method showed superior performance to several other materials and was applied for the detection of CAs quantitative detection from urine samples of Alzheimer’s disease patients.

### 2.5. Derivatization

Derivatization is an indispensable part of sample preparation in many cases, and it has been widely applied to bio-analysis [75]. In the process of trace analysis, some analytes cannot or are difficult to separate and detect directly. For example, the components to be measured have no or weak absorption in the UV-visible region; derivatization needs to be adopted at this moment. Derivatization can quantitatively transform the target compound into another compound that is easy to be analyzed and detected by using a derivatization reagent having a special functional group, and it can allow an obvious gain of the sensitivity of detection and separations of analytes, especially in complex biological samples. Moreover, derivatization can also be used to protect compounds that are unstable during the analysis process.

Catecholamine (CAs) and their metabolites are usually easy to be oxidized due to cis-diol structure and present at a low concentration level in bio-sample; sample pretreatment often applies the derivatization of the CAs and their metabolites to solve this predicament. Derivatization reagents employing acylation were frequently used in different methods. The derivatives of CAs and their metabolites possessed better chemical stability and were easier to be identified by a detector. Dansyl chloride (Dns-Cl) as a classic derivatization reagent, has been recently reported for the determination of neurotransmitters and their metabolites in plasma samples by Zhao et al. [76]. This kind of derivatization reagent has the advantages of simply derivatization operation, good derivative stability, and quantifiable reaction process; other similar reagents have also been developed like benzoyl chloride [23], 10-methyl-acridone-2-sulfonyl chloride [77], etc. However, these derivatization reagents have more or fewer limitations in their applications, some commercial derivatization reagents were also innovatively developed and applied for the derivatization of CAs and their metabolites. Ellis et al. reported a demethylation labeling method for urinary CAs and their metabolites [78]. Recently, Azaryan et al. [79] proposed a responsible approach for the determination of catecholamine in human urine using 9-fluorenyl-methoxycarbonyl chloride (FMOC-Cl). The application of FMOC-Cl can allow a complete quantitative reaction in a short time at room temperature, and this proposed procedure was successfully validated on real samples. Lissamine rhodamine B sulfonyl chloride (LRSC) was employed for the first time as a derivatization reagent for multiple NTs in LID rat brain microdialysates by Wei et al. [80]. In addition, the analyst has also designed and synthesized several derivatization reagents for CAs such as 1,3,5,7-tetramethyl-8-(N-hydroxysuccinimidyl butyricester)-difluoroboradiaza-s-indacene (TMBB-Su) [81], 4-carbonyl chloride rosamine (CCR) [29], 10-ethyl-acridone-3-sulfonyl chloride (EASC) [82], and all of them exert an excellent effect.

Our group has recently reported a novel strategy based on a combination of extraction and derivatization, coupled with LC-MS/MS analysis [83]. Zirconium oxide (ZrO_2_) nanoparticles were used for the extraction of cis-diol compounds in neutral pH conditions, and phenyl isothiocyanate (PITC) was employed as the derivatizing agent. The integration of extraction and derivatization techniques minimized the sample loss, increases the detection accuracy, and simplified the analysis. Finally, the proposed strategy was applied for the detection of CAs in healthy people and pheochromocytoma patients. Applications of derivatization for improving the sensitivity of CAs and their metabolites in biological samples are summarized in Table 3.

## 3. Analytical Techniques

**Figure 6:**
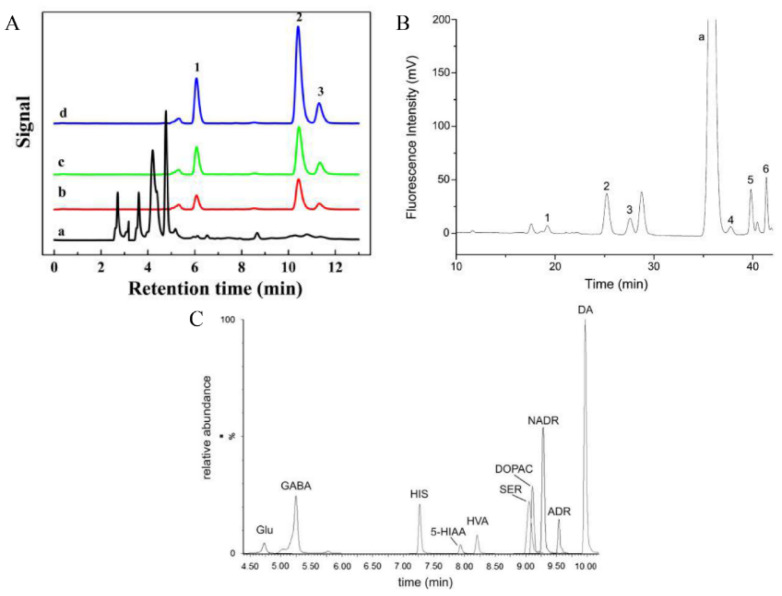
(**A**) HPLC-UV chromatograms (a), elution from B-hDIPs-SPE spiked with 0.5 μg mL^−1^ (b), spiked with 1.0 μg mL^−1^ (c), spiked with 2.0 μg mL^−1^ (d). Peaks: (1) norepinephrine, (2) epinephrine, (3) dopamine. HPLC conditions were: a Thermo scientific C18 column (250 mm × 4.6 mm, 5 mm) and UV detector at 280 nm, mobile phase, MeOH-NaH_2_PO_4_ (20 mmol L^−1^, pH 4.0) (5:95, *v*/*v*) with flow rate at 0.5 mL min^−1^ with injection volume 10 μL. Reproduced with permission from Reference [8]. (**B**) Typical chromatogram of derivatives of the six selected catecholamines and related compounds. Mobile phase: methanol and 20 mM pH 3.5 H_3_Cit–Na_2_HPO_4_ buffer. Detection: fluorescence (490/510 nm). Flow rate: 0.7 mL/min. Injection volume: 20 μL. Concentration: 0.05 μM each (L-DOPA concentration: 0.1 μM). Peaks: (1) L-DOPA; (2) Tyr; (3) NE; (4) E; (5) DA; (6) MN and (a) TMBB-Su hydrolyzate. Reproduced with permission from Reference [81]. (**C**) Extracted ion chromatogram of ten targeted analytes. Selected reaction monitoring (SRM) was used to detect targeted analytes. Chromatographic conditions: column, Acquity UPLC BEH C18 column (2.1 mm × 150 mm, 1.7 μm particle size) with VanGuard pre-column; flow rate = 0.3 mL/min; column temperature 30 °C. peaks: Glu—glutamic acid, GABA—γ-aminobutyric acid, HIS—histamine, 5-HIAA—5-hydroxyindoleacetic acid, HVA—homovanilic acid, SER—5-hydroxytryptamine, DOPAC—3,4-dihydroxyphenylacetic acid, NADR—noradrenaline, ADR—adrenaline, DA—dopamine. Reproduced with permission from Reference [23].

The measurements of CAs and their metabolites have long been an area of interest for researchers because of their important role in physiology and pathology. For decades, various detection methods have been proposed for determining the levels of CAs and their metabolites, and these analysis techniques mainly consist of high-performance liquid chromatography (HPLC) and capillary electrophoresis (CE), which are often coupled with high-performance detectors such as ultraviolet (UV) detection, fluorescence detection (FLD), laser-induced fluorescence detector (LIFD) [71], and mass spectrometry (MS). Initially, the UV detector was often introduced for the determination of CAs and their metabolites because it exhibits good stability and linearity of analysis under varying separation conditions (Figure 6A) [8,60,81,85,86]. However, by an overview of the methods for the measurements of CAs and their metabolites from 2015 to 2021 concerning CE and HPLC methods, fluorescence detection (FLD) [87] and mass spectrometry (MS) [38,88,89,90] have been more popular in comparison to UV detector. FLD is suitable for analysis of low abundance catecholamines since it has higher sensitivity and better selectivity than UV detector (Figure 6B), but due to the low fluorescence intensity of CAs and their metabolites, it needs to be introduced with the groups emitting strong fluorescence before using FLD. Later, the MS detector has risen to the forefront with the emergence of MS, increasingly taking center stage as the detection tool of choice for CAs and their metabolites. MS detection provides a lower limit of detection (LOD) and better specificity due to the identification of analytes by both retention times and molecular masses, and it can eliminate matrix interference and obtain significant gain for the determination of CAs and their metabolites, especially in complex samples. As an example, Kovac et al., [23] proposed LC-MS/MS method to identify and quantify CAs and their metabolites in cerebrospinal fluid (CSF) from the rat model for human tauopathy. Using this approach, they accurately detected changes in the concentration of CAs and their metabolites in CSF depending on the high-mass accuracy provided by the MS detector (Figure 6C). Zheng et al. [77] reported a highly sensitive strategy to detect monoamine neurotransmitters (MANTs) and their precursors and metabolites at trace levels in rat brain microdialysates. The LOQs provided by the MS detector are much lower than those provided by UV, and the combined use of various sample preparation techniques and the MS detector improves the sensitivity that can be achieved allowing the determination of CAs and their metabolites at concentrations down to 0.01 ng/mL [57].

Catecholamines and 3-methoxytyramine (3-MT) are clinically significant biomolecules and are useful for the diagnosis of phaeochromocytomas and paragangliomas (PPGLs) CAs and 3-MT are highly polar and unstable, so their determination is often limited by conventional methods. In addition, these molecules require a longer time for elution in LC-MS analysis, restricting their clinical applications [91]. Therefore, the isotope labeling strategy is very useful for detecting such compounds at the clinical level. Meiling et al. [92] developed an interesting strategy for the detection of CAs and their metabolites from plasma samples. Waters Oasis WCX μElution solid-phase extraction (SPE) was used for the extraction of metabolites, before UPLC MS/MS analysis. The whole analysis was completed in 3 min and 7 PPGLs and 408 non-PPGLs patient plasma samples were tested using the designed method. The proposed method showed high recoveries for targeted analytes ranging from 95–115% with a carryover effect of less than 10%. In addition, the ranges were 0.10–30.00 nmol/L for NE and DA, 1.00–300.00 pg/mL for 3-MT, and 05–30.00 nmol/L for E, MN, and NMN.

Chemiluminescence is another versatile technique, used for the detection of a variety of analytes from biological samples. Li et al. [93] proposed a novel, sensitive, and reliable method based on Ag (III) complex chemiluminescence detection for the analysis of monoamine neurotransmitters and metabolites including 5-hydroxypentylacetic acid (5-HIAA), levodopa (l-DOPA), epinephrine (E), 3-methoxy-4-hydroxyphenylglycol (MHPG), dopamine (DA), 3,4-dihydroxyphenylacetic acid (DOPAC), and serotonin (5-HT). From free-radical capture experiments and CL spectra, the inhibition mechanisms for luminol-[Ag(HIO_6_)_2_]^5−^-analytes CL system was investigated. Apart from several advantages for the detection of CAs and their metabolites, this method has considerably higher LODs, so trace level detection of these compounds is practically difficult. Moreover, the selectivity of the method is limited and chemiluminescence technology does not apply to all such compounds.

The in-sample ion-pairing chromatography (IPC) LC-MS/MS method is also very effective in the detection of catecholamines and their metabolites from plasma samples. In such methods, special care is needed to divert the ion-pairing reagents to waste, without entering the mass spectrometer. Marianne et al. [94] used the ion-pairing technique to quantitatively detect epinephrine (E) and norepinephrine (NE) from plasma samples with LOQ values of 0.20 and 0.02 nmol/L, respectively. Ion-pairing reagents can contaminate the ion source of the mass spectrometer, so it is mandatory to remove those before reaching the MS. In addition, ion pairs do not apply to CAs, limiting their applications in this field.

Extraction methods, coupled with electrophoretic detection, are also employed for the detection of CAa and their metabolites from biological fluids. Polikarpova et al. [95] reported a new approach based on a nano-sized polystyrene-based cation exchanger (NSCE), coated inside the capillary for the extraction and preconcentration of CAs and amino acids. The presence of surface sulfo groups efficiently separated and enriched the target compounds and their electrophoretic mobility was optimized by varying the pH. The coupling of NSCE capillaries and field-amplified sample stacking improved the detection sensitivity of the method. Designing such systems is complicated and time-consuming, and the sensitivity of the electrophoretic method is also low, compared with mass spectrometric methods.

**Figure 7:**
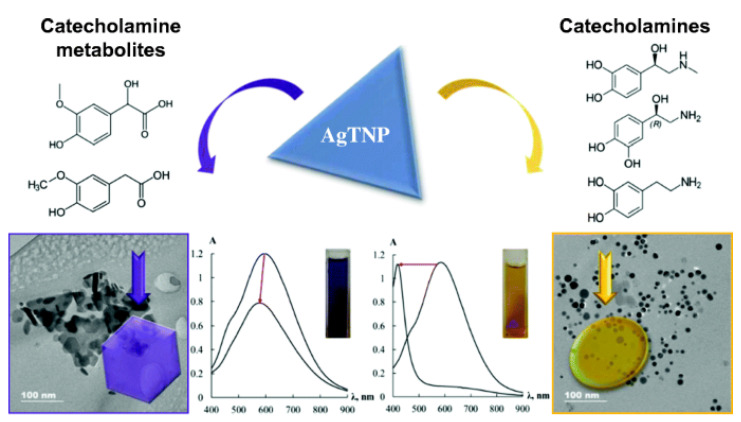
Label-free silver triangular nanoplates for spectrophotometric determination of catecholamines and their metabolites [96].

In recent research, CAs are also detected by a spectrophotometric method employing label-free silver triangular nanoplates [96]. The mechanism of detection is elaborated in Figure 7. The proposed method is simple and novel, based on the change in morphology of nanoplates, as a result, its shift to the short-wavelength region or change in local surface plasmon resonance (LSPR) band intensity is monitored. The method is rapid, selective, and sensitive to catecholamines with different functional groups.

## 4. Conclusions and Perspective

CAs and their metabolites play crucial functions in the regulation of physiological activities and the diagnosis of diseases. The current review presents an overview of recently reported sample preparation methods involved in separation-based detection techniques like LC and CE for the determination of CAs and their metabolites in biological samples. Among the reported sample preparation techniques, liquid-phase extraction has been utilized due to its simple operation and speed; however, this technique has obvious limits for the determination of CAs and their metabolites in an aqueous matrix. Luckily, SPE as an alternative has been extensively used due to its attractive advantages (compared to LLE), including the low-consuming of toxic solvents, stable recovery, and higher enrichment factors. Cation exchange, alumina, and phenylboronic acid are commonly developed for complexing CAs and their metabolites. Up until now, the traditional SPE has gradually developed into SPME, thereby, achieving higher sensitivity by using only a very small sample size. Moreover, an online extraction mode can liberate the analyst from the tedious sample preparation process, and greatly reduce the time consumed by sample preparation. At the same time, derivatization is an effective means to improve the analytical sensitivity of CAs and their metabolites. After sample preparation processes, the appropriate detection tool needs to be selected. Although UV and FLD detector has been reported, the MS detector is by far the preferred option for the detection of CAs and their metabolites. There are numerous interfering compounds in the biological sample, therefore, using an MS detector, accurate identification and quantification can be easily achieved. Moreover, with LC-MS/MS, high-throughput analysis of CAs and their metabolites is also no longer a problem.

Focusing on the determination of CAs and their metabolites, recent developments and trends for the detection of CAs and their metabolites are recognized. Conventional sample preparation techniques like PP, LLE, and SPE are still the most extensively employed for the analysis of CAs and their metabolites; however, their development needs to proceed further. Nevertheless, the automation and online connection with HPLC or CE method of these extraction techniques have not yet been fully exploited, and only a few attempts have been made to couple SPE/SPME online. It seems clear that research on the high-throughput analysis of CAs and their metabolites using online extraction coupled with MS detector will continue to be a hot topic in future.

## Figures and Tables

**Table 1:** Solid-phase extraction techniques used for extracting CAs and their metabolites in various biological samples.

Sorbents	Analytes	Matrix	LOQs	Detection	Ref.
Oasis weak cation exchange (WCX)	normetanephrine (NMN), 3-methoxytyramine (3-MT), metanephrine (MN)	human plasma	NMN: 75.0 pmol/L 3-MT: 37.5 pmol/L MN: 37.5 pmol/L	LC-MS/MS	[38]
Strata-X-CW	norepinephrine (NE), dopamine (DA), epinephrine (E)	human plasma	NE: 7.4 ng/mL DA: 5.4 ng/mL E: 3.8 ng/mL	LC-MS/MS	[39]
Evolute^®^ WCX	MN, NMN	human plasma	MN: 0.07 nmol/L NMN: 0.06 nmol/L	LC-MS/MS	[40]
96-well hydrophilic-lipophilic-balanced (HLB) Elution plate	E, NE, DA	human peripheral blood mononuclear cells (PBMC)	E: 1 pg/mL NE: 5 pg/mL DA: 5 pg/mL	LC-MS/MS	[41]
precolumn modified with phenylboronic acid	E, NE, DA	mouse urine	NE: 163 fmol/L DA: 127 fmol/L E: 196 fmol/L	LC-FLD	[42]
HLB solid-phase cartridges	E, NE, DA	human urine	NE: 0.4 ng/mL DA: 0.3 ng/mL E: 0.2 ng/mL	LC-MS/MS	[43]
Strata-X-CW	E, NE, DA, MN, NMN	urine	NE: 5.0 ng/mL DA: 5.0 ng/mL E: 5.0 ng/mL MN: 5.0 ng/mL NMN: 5.0 ng/mL	LC-MS/MS	[44]
electrospun composite fibers	E, NE, DA	human urine	NE: 0.2 ng/mL DA: 0.5 ng/mL E: 0.2 ng/mL	LC-FLD	[45]
boronate-modified hollow dummy template imprinted polymers (B-hDIPs)	E, NE, DA	human urine	NE: 157.0 ng/mL DA: 141.0 ng/mL E: 51.0 ng/mL	LC-UV	[8]
Bond-Elut Plexa	MN, NMN	urine	MN: 0.2 μmol/L NMN: 0.3 μmol/L	LC-MS/MS	[46]
96-well HLB microplate	E, NE, DA, serotonine (5-HT)	human urine	NE: 2.0 ng/mL DA: 4.0 ng/mL E: 1.0 ng/mL 5-HT: 4.0 ng/mL	LC-MS/MS	[47]
Fe_3_O_4_@PTA@MIL-100(Fe)-B	norepinephrine, epinephrine, and dopamine	Human Urine	NE: 0.050 ng/mL E: 0.11 ng/mL DA: 0.20 ng/mL	LC-FLD	[48]
magGO@POSS-BA	epinephrine, dopamine, and isoprenaline	Human urine	0.54–2.3 ng·mL^−1^	LC-FLD	[49]
Polycrown ether composite nanofiber	Catecholamines	Human urine	1 ng/mL	LC-FLD	[50]

**Table 2:** Dispersive solid-phase extraction/microextraction (DSPE/DSPME) techniques used for extracting CAs and their metabolites in various biological samples.

Sorbents	Analytes	Matrix	LOQs	Detection	Ref.
aminophenylboronic acid functionalized magnetic nanoparticles	NE, DA, E	human urine	NE: 26.0 ng/mL DA: 23.6 ng/mL E: 6.7 ng/mL	HPLC-ECD	[56]
MG@MIL-100-B composites (boronic acid functionalized MIL-100)	NE, DA, E	rat plasma	NE: 0.10 ng/mL DA: 0.01 ng/mL E: 0.01 ng/mL	HPLC-MS/MS	[57]
Fe_3_O_4_@PEI-FPBA	NE, DA, E	human urine	NE: 0.20 ng/mL DA: 0.03 ng/mL E: 0.08 ng/mL	LC-MS	[58]
CF@m-CNTs-MIP	NE, DA, E	human plasma	NE: 0.076 ng/mL DA: 0.010 ng/mL E: 0.018 ng/mL	UFLC-MS/MS	[55]
magnetic MWCNT poly(STY-DVB) composite	NE, DA, E, DL-3,4-dihydroxymandelic acid (DHMA), DL-3,4-dihydroxyphenyl glycol (DOPEG)	red deer urine	NE: 248 ng/mL DA: 205 ng/mL E: 188 ng/mL DHMA: 146 ng/mL DOPEG: 232 ng/mL	LC-MS	[59]
IDA-Cu(II) functionalized Fe_3_O_4_@SiO_2_ (Fe_3_O_4_@SiO_2_ @IDA-Cu) magnetic nanoparticles (MNPs)	NE, DA, E, 5-HT, isoprenaline(IP), tyramine (TA)	rabbit plasma	NE: 0.43 ng/mL DA: 0.20 ng/mL E: 0.33 ng/mL 5-HT: 0.16 ng/mL IP: 0.31 ng/mL TA: 0.27 ng/mL	HPLC-FLD	[19]
Fe_3_O_4_@POSS-AAPBA	NE, isoprenaline hydrochloride (IE), E	human urine	NE: 2.70 ng/mL IE: 4.40 ng/mL E: 2.96 ng/mL	HPLC-UV	[60]
Polycrown ether (PCE) composite nanofiber	biogenic monoamines	Human urine	0.25–500 ng/mL	UPLC-MS/MS	[61]
Borated zirconia	epinephrine (E), norepinephrine (NE), and dopamine (DA),	Plasma	E: 0.008 ng/mL NE: 0.020 ng/mL DA 0.004 ng/mL	LC-MS/MS	[24]

**Table 3:** Derivatization used for extracting CAs and their metabolites in various biological samples.

Derivatization Reagents	Analytes	Matrix	Derivatization Conditions			LOQs	LODs	Detection	Ref.
			T (°C)	pH	Time (min)				
10-methyl-acridone-2-sulfonyl chloride	L-DOPA, DA, NE, E, Trp, 5-HTP, 5-HT	rat brain microdialysates	37	10.5	3	0.015–0.040 nmol/L	0.002–0.010 nmol/L	UHPLC-MS/MS	[77]
TMBB-Su	Tyr, L-DOPA, DA, NE, E, and MN	mice liver and brain	25	7.6	40	--	0.10–0.40 nmol/L	HPLC-FLD	[81]
acetaldehyde	NE, DA, E, NMN, MN, 3-MT	human urine	36	5	30	1–14 nmol/L	--	LC-MS/MS	[78]
Benzoyl chloride	NE, DA, E, 5-HT, 5-hydroxyindoleacetic acid (5-HIAA), homovanilic acid (HVA), glutamic acid (Glu), γ-aminobutyric acid (GABA)	rat cerebrospinal fluid (CSF)	25	7.4	--	0.15–5.00 ng/mL	0.02–2.00 ng/mL	UHPLC-MS	[23]
FMOC-Cl	E, DA, octopamine	human urine	25	9.5	20	5–50 ng/mL	2.5–25 ng/mL	HPLC-MS/MS	[79]
methanol	20 neurochemicals	human urine	90	<4	90	0.3–12.0 ng/mL	0.1–3.6 ng/mL	UPLC-MS/MS	[84]
4-carbonyl chloride rosamine (CCR)	21 neurotransmitters (NTs)	rat brain and blood	25	9.5	1	--	1 × 10^−4^–3 × 10^−3^ nmol/L	UPLC-MS/MS	[29]
10-ethyl-acridone-3-sulfonyl chloride (EASC)	Glu, Asp, Gly, GABA, taurine (Tau), DA, 5-HT	PC12 cells	37	10.5	3	0.004–3.8 nmol/L	0.014–13.1 nmol/L	UPLC-MS/MS	[82]
Lissamine rhodamine B sulfonylchloride (LRSC)	DA, 5-HT and their biosynthesis precursors and metabolites	rat brain microdialysates	37	10.5	3	0.002–0.008 nmol/L	0.015–0.040 nmol/L	UHPLC-MS/MS	[80]
dansyl chloride	NE, DA, 5-HT, HVA, HIAA, GABA, Glu	rat plasma	65	11.0	20	--	0.991–5030 fmol/L	HPLC-MS/MS	[76]
ZrO_2_/phenyl isothiocyanate (PITC)	norepinephrine (NE), epinephrine (E) and dopamine (DA)	Human Urine	25	10	10	0.035 ng/mL	0.100 ng/mL	UHPLC-MS/MS	[83]

## Data Availability

Not applicable.
